# How Service Employees’ Mindfulness Links to Task Performance through Psychological Resilience, Deep Acting, and Customer-Oriented Behavior

**DOI:** 10.3390/bs13080657

**Published:** 2023-08-04

**Authors:** Jeong Sik Kim, Hyun Jung Park

**Affiliations:** 1 Department of Business Administration, Daejin University, Pocheon-si 11159, Republic of Korea; jskim10@daejin.ac.kr; 2 Department of International Business, Chungbuk National University, Cheongju-si 28644, Republic of Korea

**Keywords:** mindfulness, psychological resilience, customer-oriented behavior, deep acting, task performance

## Abstract

This study investigates the specific pathways through which mindfulness influences task performance, focusing on the sequential mediating roles of psychological resilience, customer-oriented behavior, and deep acting. Structural equation modeling is used to analyze data collected from 359 employees in the service industry in Korea. The results confirm that mindfulness has a significant direct and indirect relationship with task performance. Improved resilience through mindfulness can be the basis for fostering customer-oriented behavior and deep acting, which sequentially enhance task performance. This study provides a comprehensive understanding of how mindfulness leads to improvements in task performance and highlights the significance of mindfulness for both customers and service employees. It also expands the existing knowledge of mindfulness by empirically integrating resilience, customer-oriented behavior, and deep acting, which have not been extensively studied in mindfulness research. The findings have practical implications from a managerial perspective, emphasizing the importance of mindfulness resources in the workplace.

## 1. Introduction

Mindfulness, a part of Buddhist philosophy and meditation practices, is gaining attention in management as an effective means for well-being and productivity [1], attracting the interest of organizational scholars [2]. Mindfulness encompasses openness to new experiences in the present moment and the ability to let go of prior emotions or conceptions, facilitating non-judgmental interpretation, evaluation, and acceptance of experiences [3]. Mindfulness has been investigated as a one-time state involving present-centered attention and awareness [4] and as a personal trait representing a tendency to remain in a mindful state [5]. Researchers have suggested that mindfulness positively influences employee well-being [6], emotional regulation [7], job satisfaction, task performance [1,8], work engagement [9], and resilience [10,11]. Studies addressing trait mindfulness in service marketing are still in their early stages and require further investigation [12].

Previous research on mindfulness and service providers’ performance has focused on service encounter quality [12]. Lussier et al. [13] found that mindful acceptance reduces social anxiety and mitigates its negative impact on sales performance. However, there is a lack of empirical research on how mindfulness as a personal resource can be implemented in the best interests of customers, employees, and companies [12]. Enhancing employee performance through extra-role behaviors is crucial in service industries, and employee traits can influence how they interact with customers, mainly when they go the extra mile to delight them. Notably, service employees often perform emotional labor, defined as an act in which they control and express emotions in a way that is consistent with social expectations in the service field [14].

Emotional labor involves managing emotions to modify emotional expressions, and coping strategies can be surface or deep acting [15]. Deep acting is based on authenticity or attempts to change internal feelings and genuine efforts to experience the required emotions, whereas surface acting involves displaying the required emotions with facial expression, tone, and gesture while keeping one’s genuine emotions unchanged [14,16]. Whereas surface acting drains energy, induces negative emotions and interferes with social interactions, deep acting prevents resource depletion and discrepancies between felt and expressed emotions [17,18]. Consequently, deep acting can help reduce emotional dissonance, leading to heartfelt service [19].

Researchers emphasize the need for studies on reducing surface acting and promoting healthier coping strategies for emotional job demands [7]. The findings indicate that mindfulness affects surface acting and emotional exhaustion [7] and that manipulating state mindfulness improves service quality [12]. However, those with induced state mindfulness experience more emotional labor, contrary to the expectation that they would display more of their true feelings. Consequently, more research is required to understand how mindfulness affects service employees’ psychological states and how its influence differs between one-off mindfulness and trait mindfulness [12]. Previous research has highlighted the direct relationship between mindfulness and its desirable outcomes without delving into the underlying processes. Thus, this study aims to uncover the specific pathways through which service employees’ mindfulness affects their task performance. 

As a specific path, we focus on the impact of service employees’ extra efforts and voluntary actions, such as deep acting and customer-oriented behavior, on customer performance. Deep acting requires energy investment prior to emotional encounters [17] or cognitive effort to regulate emotional experiences [18], highlighting the need for more psychological resources. Customer-oriented behavior, a key predictor of task performance in the service industry [20], focuses on satisfying customer needs through active engagement [21]. As no studies have shown the positive effects of mindfulness on these behaviors, we propose that psychological resilience explains these relationships. According to the conservation of resources (COR) theory [22], additional efforts require energy, and resilience may help employees put more effort into better service.

Psychological resilience is the ability to return to one’s initial state after experiencing hardship, conflict, or failure [23]. It has been studied as an essential antecedent that helps resolve stressful situations and prevent burnout [24,25,26]. It gained attention during the COVID-19 pandemic, as resilient employees show better adaptability, engagement, and performance [27,28]. Building a resilient workforce is crucial for gaining a competitive edge through improved service behavior, particularly for frontline personnel who have contact with customers [27]. They often encounter adverse events associated with customers, and their psychological recovery is crucial for improved emotional performance [29]. 

In summary, resilience facilitates the acquisition of the external resources required to provide high-level service [29,30], which predicts the task performance of employees who have contact with customers [20,31]. Mindfulness helps alleviate employees’ emotional exhaustion and enhances their resilience [32,33]. These findings support the possibility of serial mediation in which resilience, customer-oriented behaviors, and deep acting play crucial roles in explaining the process from mindfulness to task performance. 

To the best of our knowledge, no empirical research has explored whether employees’ trait mindfulness leads to better and more voluntary interactions with customers or how deep acting and customer-oriented behaviors together affect task performance. To fill this gap, the present study investigates the impact of mindfulness on deep acting, customer-oriented behavior, and task performance. We also examine the combined effects of customer-oriented behaviors and deep acting on task performance while exploring the mediating role of resilience between mindfulness and the two constructs that can contribute to task performance.

## 2. Theoretical Framework

### 2.1. Mindfulness, Task Performance, and Resilience

Trait mindfulness is a dispositional characteristic encompassing self-awareness, a present-oriented mindset, and nonjudgmental observation [5]. It has received significant attention in organizational psychology and management because of its various positive outcomes for employees [34,35]. According to a meta-analysis, trait mindfulness is positively related to employee confidence, mental health, emotional regulation, job satisfaction, and performance. It is negatively associated with stress, anxiety, burnout, and depression, indicating its potential to promote well-being and reduce negative psychological states [36]. 

Mindful employees can focus on the present and objectively control their thoughts and emotions concerning their work, as mindfulness allows them to assess events more objectively and effectively regulate their thoughts and feelings [37]. They concentrate on their work’s immediate needs and separate themselves from work events, and emotions felt [34], benefiting from attention to the present moment when coping with demanding situations [4]. As the ability to recognize environmental cues and internal states may foster mental balance, individuals are more likely to feel in control under challenging circumstances, thereby experiencing competence and autonomy [7]. The attention and acceptance aspects of mindfulness decrease anxiety and stress and improve task performance related to emotion regulation [38].

Mindfulness has been associated with lower emotional exhaustion and higher psychological capital across various occupations [6]. Cultivating mindfulness can help individuals cope better with challenges and enhance their ability to bounce back from setbacks. A reflective state and focus on current experiences can provide psychological resources to deal with and overcome stressful experiences and decrease burnout [39]. Therefore, mindfulness is related to elements of psychological capital, primarily resilience [32]. Psychological resilience refers to the tendency to actively adjust to stress and the crucial personal capacity to respond appropriately to uncomfortable events [40], relieve job stress, and trigger desirable behaviors through motivation and positive cognitive status [41]. This can be improved by reducing stress or preventing burnout [10,11]. 

As a personal resource, mindfulness promotes positive coping and resilience to stress [33] by drawing individuals into the present moment to assist them in perceiving greater control over events [34]. It contributes to resilience by helping individuals reduce habitual worry and directing their attention toward finding solutions rather than focusing solely on adversities [42]. It also motivates employees to maintain vitality by heightening their wakefulness and involvement [9]. According to the mindfulness-to-meaning theory [43], mindfulness engenders flexibility in eudemonic meaning creation, enhancing a person’s ability to reappraise adverse experiences and savor more positive aspects. Promoting positive emotions and reappraisal may stimulate meaning in work, which fosters resilience and performance. Accordingly, we hypothesize as follows.

**H1.**  *Service employees’ mindfulness is positively associated with their task performance.*

**H2.**  *Service employees’ mindfulness is positively associated with their psychological resilience.*

### 2.2. Resilience, Customer-Oriented Behavior, and Deep Acting

The COR theory suggests that an individual’s resource level is related to resource allocation behavior [22]. Those with sufficient resources to deal with a stressor or challenge experience less stress can tolerate resource loss, and prefer strategies that potentially acquire resources. By contrast, those with fewer resources are more susceptible to resource loss and tend to conserve resources. Employees needing more resources owing to low resilience tend to save their remaining resources and detach themselves from work. By contrast, those with high resilience are more likely to be committed and engaged. Resilient employees gain external resources for high-quality services [29,30] and solve problems more efficiently by creating positive emotions [44]. As psychological resilience is a crucial ability to respond positively to uncomfortable situations [40], it is a critical personal resource for service employees who have contact with customers to decrease their stress and promote service orientation and deep acting [29]. 

Customer-facing tasks that can damage the subjective well-being of emotional workers [17] emphasize the importance of psychological resilience. The continuous monitoring of disguised emotions while suppressing genuine inner emotions is accompanied by a significant cognitive burden, resulting in stress and exhaustion [45]. Individuals with a high level of customer orientation attempt to achieve customer satisfaction by establishing long-term relationships with customers [21]. However, inconsistency between actual emotions and behaviors leading to mental conflict or emotional dissonance depletes psychological resources, causing insensitive or cynical responses to customers, a decreased sense of achievement, and job burnout [45,46]. 

Deep acting is an emotional strategy that demands more significant psychological resources and is more challenging to achieve than surface acting [47] owing to the cognitive transformation efforts required to recognize one’s feelings about the job situation and to control them to genuinely feel more appropriate emotions. Deep acting aims to genuinely experience and express organizationally required emotions instead of superficially displaying them to customers [16]. This is an active attempt to change internal emotions rather than simply modifying external emotions [16]. Whereas surface acting is likely to be triggered in typical behavioral patterns, such as automatic smiling, deep acting accompanies the conscious process of regulating behavior to actively influence one’s feelings. This requires additional psychological resources [48]. Surface acting involves emotion control efforts to suppress emotions felt after service performance and to express artificial emotions. By contrast, deep acting requires preliminary control efforts to internalize emotional expression rules before performing emotional labor [16].

According to the COR theory, the more aware emotional workers are of available resources owing to resilience, the more likely they are to invest their immediate resources and direct their psychological energy to active services, focusing on potential gains rather than the loss of resources. Deep acting can increase resources by improving performance. Thus, employees with more personal resources are less likely to view deep acting as a threat to their resource retention goals, even if it immediately consumes more resources than surface acting [49]. Those who perceive encounters with aggressive customers as stressful tend to use surface acting to avoid resource loss, whereas those who view such encounters as less stressful spend resources on deep acting [49]. The enthusiastic attitude of workers to deliver services to customers and express positive feelings from within is closely related to resilience; according to a meta-analysis related to trait resilience and mental health, resilience reduces anxiety, depression, and negative emotions and improves positive emotions, such as life satisfaction [50]. Although the persistent tension between job stress and an environment in which customers must be provided with satisfying services often leads to exhaustion [45], resilience may encourage professional activities that identify, analyze, understand, and satisfy customer needs [51], promoting personal goals that drive employees to meet customers’ needs. Therefore, the more resilient employees are, the more likely they are to engage in customer-oriented behavior and deep acting.

**H3.**  *Psychological resilience is positively associated with customer-oriented behavior.*

**H4.**  *Psychological resilience is positively associated with deep acting.*

### 2.3. Customer-Oriented Behavior, Deep Acting, and Task Performance

In a rapidly changing business environment, customer orientation that actively responds to customer needs is essential. The essence of customer orientation is to avoid sales-centric behaviors that jeopardize customer interests and aid customers in accurately understanding their needs and making optimal purchasing decisions [52]. Customer orientation is positively correlated with customer satisfaction [53], service quality perception [54], and sales performance [55]. Customer orientation may derive higher levels of customer satisfaction by discovering or fulfilling customer needs. Enhancing customer evaluation of service quality is tied to the task performance of service personnel [31].

**H5.**  *Customer-oriented behavior is positively correlated with task performance.*

While surface acting amplifies emotional dissonance and negatively affects job attitudes and performance, deep acting positively impacts individuals’ psychological and physical health [49] and enhances customer satisfaction and task performance [16]. 

Emotional dissonance can be minimized when behaviors that elicit responses, which should be expressed externally, become routine [45]. Deep acting can prevent job burnout by increasing the sense of efficacy and achievement without compromising emotional authenticity [56], promoting positive customer relationships. Consequently, it can improve customer service quality and task performance [17,47,57].

**H6.**  *Deep acting is positively correlated with task performance.*

### 2.4. Sequential Mediating Role of Psychological Resilience, Customer-Oriented Behavior, and Deep Acting

However, the impact of mindfulness on service providers’ resilience and emotional labor has not yet been investigated. Mindfulness may indirectly influence customer-oriented behavior or deep acting and, ultimately, task performance by increasing psychological resilience. Increased resilience creates a positive cognitive state and provides sufficient motivation to create situations in which desirable behaviors are achieved [41]. Paying attention to and maintaining a close relationship with customers leads to customer-oriented behavior and deep acting through proactive attempts to change internal emotions. 

Service employees’ customer-oriented behavior and deep acting help them understand customer needs and assist customers in making optimal purchasing decisions [52]. This raises the awareness of service quality [54] and customer satisfaction [53]. Employees can enhance service quality and achieve superior performance by deep acting in situations that demand emotional labor [17,47,57]. To sequentially link mindfulness and performance, we propose the following hypothesis.

**H7.**  *Psychological resilience, customer-oriented behavior, and deep acting sequentially mediate the relationship between mindfulness and task performance.*

**Figure 1:**
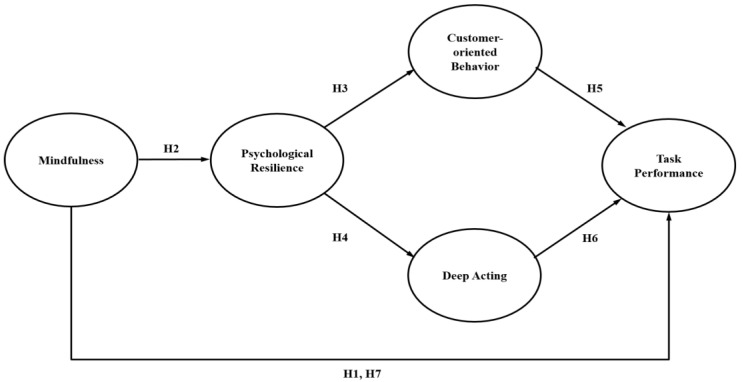
Research model.

The seven hypotheses are shown in Figure 1.

## 3. Methods

### 3.1. Participants

This study conducted a survey targeting 400 employees working for 20 companies listed on the Korean stock market from June to July 2022, lasting for 2 months. All employees were in service occupations. The survey was conducted by research assistants who visited the companies and collected data directly. The main survey targets were subordinate employees. To ensure objectivity in measurement and mitigate self-serving bias, immediate supervisors were requested to respond directly to task performance measurements. Before conducting the survey, the participants were given clear explanations about the purpose of the study. It should be noted that the collected data will be used for research purposes only, treated anonymously, and kept strictly confidential. Consent to use the research data was obtained from all the respondents. When collecting the survey forms, the research assistants integrated the completed surveys from the respondents and the task performance measurement surveys conducted by the immediate supervisors into a prepared envelope, which was then sealed. 

After excluding incomplete questionnaires, 359 valid questionnaires were obtained, corresponding to an 89.5% response rate. Table 1 summarizes the sample profiles of respondents. From a sociodemographic perspective, the respondents included 210 men (58.5%) and 149 women (41.5%). Married individuals accounted for 273 (76.0%), which was significantly higher than the proportion of single individuals (86 [24.0%]). An overwhelming proportion of the respondents (97.4%) held a university degree. The average tenure within the organization was 12.8 years. The average department size was 9.6 members.

### 3.2. Measures

All questionnaire items were designed on a 5-point Likert scale. Measures are presented in Table 2.

Mindfulness was measured by adapting and reconstructing six items from Brown and Ryan’s [5] scale. Sample items included “I pay attention to what is happening” and “I tend to work while always aware of what I am doing”.

For psychological resilience, we adopted six items from Luthans et al. [58], such as “When I have a setback at work, I don’t have trouble recovering from it, moving on” and “I can be “on my own”, so to speak, at work if I have to”.

Customer-oriented behavior was assessed using Stock and Hoyer’s [59] six items, including “I try to discuss customers’ needs” and “I try to influence customers through information rather than pressure”.

Deep acting was measured using three items taken from Brotheridge and Lee [60]. The sample items included “I just try to experience emotions that need to be shown to customers in actual relationships with them” and “I express emotions that need to be revealed as a part of my duty through actual feelings in relationship with customers”.

For employee performance, we asked supervisors to complete seven items taken from Williams and Anderson [61]. The supervisors indicated how much they agreed with such statements as, “My subordinate … fulfills the responsibilities specified in the job description” and “My subordinate … meets the formal performance requirements of the job”.

## 4. Results

### Reliability and Validity Test

Table 3 presents the confirmatory factor analysis (CFA) results. The baseline five-factor model demonstrates a good fit with the data (*χ*^2^ = 805.07, *df* = 340, *p* < 0.01; CFI = 0.93, TLI = 0.92, SRMR = 0.05, RMSEA = 0.06). The criteria for goodness-of-fit indexes are as follows: CFI and TLI should be 0.90 or lower, SRMR should be 0.80 or lower, and RMSEA should be 0.70 or lower [62]. Based on these criteria, the CFA model used in this study is acceptable. For comparison purposes, we test four alternative models against this baseline five-factor model. Model 1 is a four-factor model that combines customer-oriented behavior and deep acting into a single factor; Model 2 combines customer-oriented behavior, deep acting, and task performance into one factor; Model 3 is a two-factor model that combines mindfulness and psychological resilience into one factor, and customer-oriented behavior, deep acting, and task performance into another; and Model 4 merges all items into a single factor. The fit indexes support the hypothesized five-factor model, confirming the distinctiveness of the constructs.

As shown in Table 4, all factor loadings of the variables exceed the criterion of 0.50, and the average variance extracted (AVE) is also above 0.50, ensuring convergent validity [62]. Discriminant validity is analyzed by comparing the square roots of the AVE and correlation coefficients, as shown in Table 5. Discriminant validity is also confirmed, as the square root of the AVE for each variable is higher than the correlation coefficients with other variables [63]. Moreover, composite reliability exceeds the criterion of 0.70, ensuring reliability [62].

The mean values of the constructs range from 3.59 to 3.89, with standard deviations ranging from 0.62 to 0.67. Deep acting had the lowest mean, whereas task performance had the highest. All constructs exhibit consistent distributions, with standard deviations below 1.0, indicating a consistent pattern. Structural equation modeling (SEM) with AMOS 23.0 is employed to test the hypotheses. The overall fit index (*χ*^2^ = 891.35, *df* = 344, CFI = 0.92, TLI = 0.91, RMSEA = 0.07, SRMR = 0.07) suggests that the model accurately represents the structure underlying the observed data.

**Figure 2:**
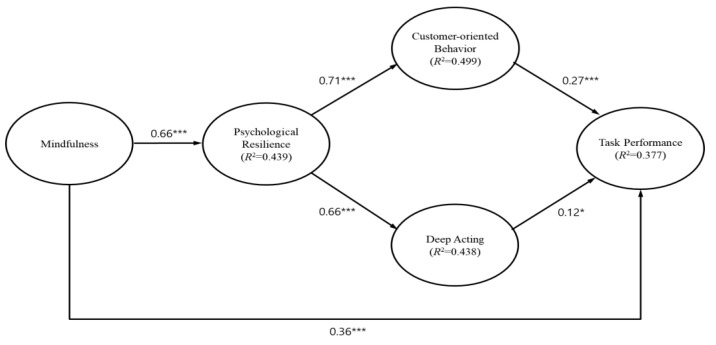
Results of structural equation modeling analysis. Notes: * *p* < 0.10, *** *p* < 0.01.

Based on the analysis results presented in Figure 2, the following hypotheses are tested: mindfulness is positively related to task performance (*β* = 0.36, *p* < 0.01), supporting H1. Mindfulness enhances psychological resilience (*β* = 0.66, *p* < 0.01), supporting H2. Both H3 and H4 are supported, as psychological resilience is found to increase customer-oriented behavior (*β* = 0.71, *p* < 0.01) and deep acting (*β* = 0.66, *p* < 0.01). Given that customer-oriented behavior is associated with task performance (*β* = 0.27, *p* < 0.01), H5 is supported. Deep acting is also related to task performance (*β* = 0.12, *p* < 0.10), supporting H6. The statistical significance of the indirect effects is tested using bootstrapping in AMOS. The analysis in this study is conducted with a 99% confidence interval. The criterion for determining significance is that zero should not be included between the lower and upper bounds of the analysis results [64]. According to the results, the indirect effect through the three mediators (psychological resilience, customer-oriented behavior, and deep acting) is statistically significant (*β* = 0.13, *p* < 0.01), as zero is not included between the lower and upper bounds (99% CI = [0.10, 0.38]). Therefore, H7 is supported.

Additionally, we include the path from psychological resilience to task performance, and the result is not significant (*β* = 0.04, *p* > 0.10), indicating an indirect effect of psychological resilience on task performance.

## 5. Discussion

This study investigated how trait mindfulness of employees working at customer contact points affects task performance. We tested trait mindfulness and incorporated psychological resilience as a missing link, suggesting that improved psychological resilience through mindfulness could foster customer-oriented behavior and deep acting, ultimately boosting task performance. We examined whether psychological resilience, customer-oriented behavior, and deep acting sequentially explain this mechanism. The results are as follows: Employee mindfulness was directly linked to task performance and indirectly linked to task performance through psychological resilience, customer-oriented behavior, and deep acting. Second, mindfulness positively affected psychological resilience, suggesting that it can provide an opportunity to recharge energy for more proactive work. Psychological resilience enhanced customer-oriented behavior and deep acting. Third, customer-oriented behavior and deep acting positively impacted task performance, and customer-oriented behavior had a stronger influence than deep acting.

### 5.1. Theoretical Implications

By demonstrating the role of mindfulness in restoring workers’ psychological resources and promoting positive efforts for customers, this study contributes to the existing knowledge of mindfulness in service marketing.

First, it expands the research on mindfulness by adding empirical evidence and exploring the pathways through which mindfulness influences task performance. Our results are consistent with previous studies [1,8] that mindfulness enhances task performance. Furthermore, we contribute to the literature by suggesting a mechanism that needs to be examined. This study presents and validates psychological resilience, customer-oriented behavior, and deep acting as sequential mediators in the pathway from mindfulness to task performance. These findings highlight the significance of psychological resilience as a critical psychological state for employee motivation and proactive actions for customers. The results are especially significant, as they demonstrate a more refined and logical approach that has not been addressed in previous research.

Second, this study confirms the influence of mindfulness in emotional labor and service marketing contexts by considering customer–employee interactions. Employees who engage in customer-oriented behavior tend to think more, pay more attention, and work harder to identify, understand, and satisfy customer needs [51]; deep acting requires cognitive effort and energy investment [17]. Mindful employees may effectively tune their thoughts and emotions by paying attention to their experiences and recognizing the overall context [37]. Thus, the efficient distribution and use of mental energy may prevent exhaustion and restore vigor to elicit more genuine and sincere behaviors during emotional labor. Given that little is known about the psychological resilience mediating the relationship between mindfulness and emotional labor, this study introduced the influence of mindfulness on psychological resilience in a service marketing context.

Third, the results highlight the importance of psychological resilience, which is essential for linking psychological traits and positive attitudes toward an organization [65]. This study expands research on the role of psychological resilience in emotional labor strategy selection by confirming that psychological resilience can enhance consumer-oriented behavior and deep acting. Researchers have identified several individual characteristics, including emotional intelligence [66], psychological capital [67], and personality traits [68], as antecedents of emotional labor strategy selection. Our results indicate that psychological resilience is crucial for responding to customers, exploring solutions to problems, and providing value in challenging service environments. Flexible adaptation to changing needs enables employees to grow more by maintaining and restoring psychological energy in difficult situations [23]. Those with higher levels of psychological resilience to restore vitality, even with negative experiences, may have proactive job attitudes and make efforts to provide quality services.

### 5.2. Practical Implications

The findings offer managerial insights into the importance of mindfulness resources in the workplace. First, companies striving to provide high-quality services should consider mindfulness and psychological resilience when recruiting potential high performers and foster these psychological resources through training and education. Employee psychological resilience is flexible and can be enhanced through practical training and development initiatives [27,28]. Although innate, mindfulness is an individual characteristic that undergoes dynamic changes over time and is influenced by ongoing interactions with environmental factors. Cultivating psychological resources can help employees better understand and manage their emotions, enabling them to genuinely engage in deep acting and show sincere concern for customer needs. For those working in service-oriented positions, it is vital to recognize the importance of mindfulness and psychological resilience and make efforts to maintain them. This involves adopting a present-focused mindset, incorporating self-reflection and self-awareness practices into daily routines, and working actively to recover from adversity and challenges [25].

Second, when evaluating the performance of service-sector employees, it is necessary to consider not only task performance outcomes but also employees’ customer orientation and emotional labor strategies associated with the service delivery process. Such efforts are likely to encourage service personnel to pursue customer satisfaction and establish long-term customer relationships. Finally, customer-oriented behavior has a more substantial influence on performance than deep acting, which may be attributed to the fact that customer-oriented behavior encourages purchases by building relationships with customers or catering to their needs, while deep acting centers around genuinely expressing positive emotions. Deep acting remains essential and contributes to job satisfaction and organizational commitment [69].

### 5.3. Limitations and Future Research

There are some limitations and potential avenues for future research. 

Although we attempted to minimize self-serving bias by having supervisors measure subordinates’ task performance directly, potential biases in other relationships among the variables cannot be ruled out. Future research could consider a longitudinal design instead of a cross-sectional one to capture the influence of mindfulness over time. The influence of mindfulness can be accurately measured if multiple measurements are performed over a certain period. Studies on workers have focused on the tendency to be mindful (trait or dispositional mindfulness), while others have investigated the role of meditation (mindfulness interventions) and state of mind (state mindfulness). 

Future studies should incorporate other psychological variables related to resource conservation and mindfulness. Based on the COR theory, individual traits, such as emotional intelligence and extraversion [70], may mitigate the loss of psychological resources due to emotional labor effort [29]. Future studies should apply attributes of organizational effectiveness beyond task performance, such as creativity, innovation, and personal capacity growth, which would offer broader implications. 

## Figures and Tables

**Table 1:** Demographic characteristics of the respondents.

Variable	Category	Frequency	Percentage (%)
Gender	Women	269	74.9
	Men	90	25.1
Age (years)	30–39	48	13.4
	40–49	272	75.7
	50–59	33	9.2
	Above 60	6	1.7
Marital Status	Single	86	24.0
	Married	273	76.0
Education Level	High School Graduate	9	2.5
	University Graduate	189	52.6
	Graduate School Graduate	161	44.8
Tenure (years)	Below 10	78	21.7
	10–20	242	67.4
	Above 20	39	10.9

**Table 2:** Questionnaires and sources.

Variable	Questionnaire	Source
Mindfulness (six items)	I pay attention to what is happening. I tend to work while always being aware of what I am doing.	[5]
	I pay attention in advance when engaging in certain activities.	
	I handle tasks with an awareness of what I am currently doing.	
	I am not caught up in the past or the future. I find myself doing things with paying attention.	
Psychological resilience (six items)	When I have a setback at work, I don’t have trouble recovering from it, moving on.	[58]
	I usually manage difficulties one way or another at work.	
	I can be “on my own”, so to speak, at work if I have to.	
	I usually take stressful things at work in stride.	
	I can get through difficult times at work because I’ve experienced difficulty before.	
	I feel I can handle many things at a time in this job.	
Customer-oriented behavior (six items)	I try to discuss customers’ needs.	[59]
	I answer customers’ questions about products and/or services as correctly as I can.	
	I try to influence customers through information rather than pressure.	
	I try to give customers an accurate expectation of what the product will do for them.	
	I am willing to disagree with the customers to help them make better decisions.	
	I try to help customers to achieve their goals.	
Deep acting (three items)	I just express emotions to customers through efforts for actual experience.	[60]
	I just try to experience emotions that need to be shown to customers in actual relationships with them.	
	I express emotions that need to be revealed as a part of my duty through actual feelings in relationships with customers.	
Task performance (seven items)	My subordinate … adequately completes assigned duties.	[61]
	My subordinate … fulfills the responsibilities specified in the job description.	
	My subordinate … performs tasks that are expected of them.	
	My subordinate … meets the formal performance requirements of the job.	
	My subordinate … engages in activities that will directly affect their performance evaluation.	
	My subordinate … doesn’t neglect aspects of the job they are obliged to perform.	

**Table 3:** Results of confirmatory factor analysis.

Model	Factors	*χ* ^2^	Δ*χ*^2^	*df*	Δ*df*	*χ*^2^/*df*	CFI	TLI	RMSEA
Null model		6795.50		378					
Baseline model	Five factors.	805.07		340		2.37	0.93	0.92	0.06
Model 1	Four factors: Customer-oriented behavior and deep acting, are combined into one factor.	965.00	159.93 **	344	4	2.81	0.90	0.89	0.07
Model 2	Three factors: Customer-oriented behavior, deep acting, and task performance are combined into one factor.	1859.13	1054.06 **	347	7	5.36	0.76	0.74	0.11
Model 3	Two factors: Mindfulness and psychological resilience, are combined into one factor; customer-oriented behavior, deep acting, and task performance are combined into another factor.	2297.92	1492.85 **	349	9	4.26	0.70	0.67	0.13
Model 4	One factor: All items are combined into one factor.	2779.00	1973.93 **	350	10	7.25	0.81	0.85	0.17

Notes. *χ*^2^ values for the measurement and structural models are significant at *p* < 0.001. All Δ*χ*^2^ and Δ*df* values are about the baseline model. ** *p* < 0.01.

**Table 4:** Measurement properties.

Construct		Standardized Loadings	Reliability	Variance-Extracted Estimate
Mindfulness	×1	0.75	0.87	0.61
	×2	0.80		
	×3	0.80		
	×4	0.66		
	×5	0.72		
	×6	0.66		
Psychological resilience	×1	0.68	0.89	0.64
	×2	0.77		
	×3	0.79		
	×4	0.80		
	×5	0.67		
	×6	0.80		
Customer- oriented behavior	×1	0.81	0.89	0.64
	×2	0.76		
	×3	0.78		
	×4	0.81		
	×5	0.64		
	×6	0.76		
Deep acting	×1	0.73	0.80	0.64
	×2	0.76		
	×3	0.78		
Task performance	×1	0.86	0.94	0.73
	×2	0.83		
	×3	0.83		
	×4	0.83		
	×5	0.84		
	×6	0.81		
	×7	0.82		

**Table 5:** Means, standard deviations, and correlations.

Construct	Mean	S.D.	Correlation Coefficients				
			1	2	3	4	5
Mindfulness	3.72	0.60	(0.78)				
Psychological resilience	3.70	0.63	0.56 **	(0.80)			
Customer- oriented behavior	3.74	0.62	0.56 **	0.60 **	(0.80)		
Deep acting	3.59	0.65	0.53 **	0.52 **	0.58 **	(0.80)	
Task performance	3.89	0.67	0.52 **	0.45 **	0.51 **	0.43 **	(0.85)

Note: AVE square roots are in parentheses along the diagonal. ** *p* < 0.01.

## Data Availability

The data used to support the findings of this study are available from the corresponding author upon request.
